# Functional Role of SIL1 in Neurodevelopment and Learning

**DOI:** 10.1155/2019/9653024

**Published:** 2019-08-18

**Authors:** Shilian Xu, Jia Zhu, Kai Mi, Yan Shen, Xiaomin Zhang

**Affiliations:** ^1^Department of Physiology, School of Basic Medicine, Kunming Medical University, Kunming, Yunnan Province, China; ^2^Department of Pathology, Institute of Diabetes and Nerve Disease, Jiaxing Hospital of Traditional Chinese Medicine, Jiaxing University, Jiaxing, Zhejiang Province, China

## Abstract

**Background:**

*Sil1* is the causative gene of Marinesco-Sjӧgren Syndrome (MSS). The mutated *Sil1* generates shortened SIL1 protein which will form aggregation and be degraded rapidly. Mental retardation is a major symptom of MSS which suggests a role of SIL1 in the development of the central nervous system, but how SIL1 functions remains unclear.

**Objectives:**

The aim of this study is to explore the role of SIL1 in regulating cerebral development and its underlying molecular mechanism.

**Methods:**

The basic expression pattern of SIL1 in tissues and cultured cortical neurons is measured by immunostaining and Western blot. The expression of SIL1 is reduced *in vitro* and *in vivo* through RNA interference delivered by a lentivirus. The expression of NMDA receptor subunits and the function of the Reelin signaling pathway are then examined by surface biotinylation and Western blot subsequently. Finally, the spatial learning of young mice was assessed by the Barnes maze task.

**Results:**

SIL1 deficiency caused a diminished expression of both Reelin receptors and therefore impaired the Reelin signaling pathway. It then inhibited the developmental expression of GluN2A and impaired the spatial learning of 5-week-old mice.

**Conclusions:**

These results suggested that SIL1 is required for the development of the central nervous system which is associated with its role in Reelin signaling.

## 1. Introduction

SIL1 is an Endoplasmic Reticulum- (ER-) resident 54 kD protein that is composed of 461 amino acids [1]. SIL1 has an ER-targeting sequence in its amino terminus and an ER retention KDEL sequence in its carboxyl terminus [2]. SIL1 is the mammalian homolog of yeast Sls1p/Sil1p and functions as the nucleotide exchange factor of ER chaperone protein Bip [3]. It is well acknowledged that Bip is a member of the heat shock protein 70 family and it plays important roles in mediating folding and assembly of nascent proteins, as well as degradation of misfolded proteins [4–6]. Both binding and separating of Bip from its substrate protein require the assistance of cofactors [2]. As an adenine nucleotide exchange factor, SIL1 regulates the ATPase activity of Bip and promotes the release of the substrate protein [3, 7].

In 2005, two groups independently identified SIL1 (gene ID: 64374) as the causative gene of Marinesco-Sjӧgren Syndrome (MSS; OMIM 248800) [8, 9]. Single-gene mutation of SIL1 is enough to cause MSS. MSS is an autosomal recessive multisystem disorder, and its main symptoms include cerebellar ataxia, cataracts, mental retardation, myopathy, and short stature [9–11]. Different kinds of SIL1 mutation have been discovered in MSS patients, including missense mutation, in-frame deletion, and several single-nucleotide mutations that affect RNA-splicing sites [11–14]. These mutations cause codon shift or deletion inside exon 6 and exon 9 which result in abnormal expression of the protein [15]. As a consequence, the mutated SIL1 that could not bind with Bip or be stably retained in ER would be transported into the cytoplasm and degraded by the proteasome [12, 16].

As a cofactor of Bip, SIL1 expresses in all types of cells; however, only several organs are affected in MSS, especially the central nervous system. 90% of the MSS patients showed moderate to severe mental retardation [11], which indicates that SIL1 may play specific roles in the nervous system. It has been proved that the SIL1-mutated *woozy* mice developed ataxia which resulted from Purkinje cell loss in the anterior cerebellar lobules [17]. A recent research found that the migration and morphological maturation of cortical neurons during development are impaired after RNAi mediated gene silencing of SIL1. However, the cortical localization of neurons in adult mice was normal which suggested that the migration was only delayed but not irreversibly inhibited. These studies showed that SIL1 plays important roles in the development of the mammalian cortex and cerebellum, but the mechanism is still not clear yet. Furthermore, current evidence could not directly prove that these developmental defects caused by SIL1 deficiency are responsible for the intelligent disability of MSS patients. In our study, we aimed to provide new insights regarding these issues.

In our former study, we found that Bip was involved in the dynamic trafficking of the GluN2A-containing NMDA receptor induced by plasticity stimulation and the subunit-selective interaction between Bip and GluN2A was required for fear conditioning [18]. The NMDA receptor is a crucial excitatory receptor in the central nervous system which is a heterogeneous tetramer and has several subtypes. During early development, the GluN2B-containing NMDA receptor is the dominant subtype in the frontal cortex and then GluN2A increases extensively after birth and becomes the predominant subunit. This GluN2B to GluN2A dominancy shift is crucial for the intellectual development of mammals. Since SIL1 is the cofactor of Bip, we speculated that SIL1 may also be involved in the trafficking of GluN2A. Therefore, we inhibited SIL1 expression through RNA interference both *in vitro* and *in vivo*, and we found that the postnatal expression of the GluN2A-containing NMDA receptor was inhibited and the spatial learning of mice was impaired, both of which are caused by the impaired Reelin signaling pathway. Therefore, our data proved for the first time that SIL1 regulates the expression of GluN2A through the extracellular matrix-dependent signaling pathway which is essential for the neurodevelopment and spatial memory formation.

## 2. Materials and Methods

### 2.1. Animals

Male and female C57 mice were obtained and acclimated to the colony room for at least 2 weeks prior to mating. The colony room was maintained on a 14 hr light/10 h dark cycle with controlled temperature and humidity with food and water available freely. The subjects were the offspring from the pairing of a female with a male partner. Virus was injected at 3 weeks old and behavioral task was performed 2 weeks later.

### 2.2. Cortical Neuron Culture

For the primary neuron culture, we followed the methods of Zhang et al. [18]. Cortical neuronal cultures were prepared from one-day-old mice. Briefly, the cortex tissue was extracted and chopped to be 1 mm∗1 mm size and then digested in 0.28% trypsin (Sigma, St. Louis, MO) for 15 min at 37°C with gentle shaking. Then, the sample was rinsed twice with ECS, and then the dissociated cells were plated at a density of 2 × 10^6^ in a 35 mm (for staining) dish on poly-L-lysine-coated coverslips or directly in a 60 mm dish. 2 ml or 4 ml of neurobasal plus medium (Invitrogen, Carlsbad, CA) containing 10% FBS, 2 mM glutamine, and 100 *μ*g/ml gentamycin (all from Invitrogen Corporation, Carlsbad, CA) was added, and then cells were maintained at 37°C in 5% CO_2_. After 24 hr, the medium was replaced with neurobasal medium containing 2% B27 supplement, 1% antibiotic, and 0.25% glutamine (Invitrogen). At DIV5, cytosine arabinofuranoside was added at a final concentration of 10 *μ*M. Thereafter, half of the medium was replaced twice a week.

### 2.3. RNA Interference

The lentivirus of shRNA was designed to target 2 distinct coding sequences in mouse SIL1 according to a former literature [19] (shRNA#1: 5′-GCTCCAACAAGAAGACAAA-3′; shRNA#2: 5′GGTTGCTGCGCTCTTTGAT-3′) (constructed by GenePharma, Suzhou, China). For the cultured cortical neurons, shRNA#1, shRNA#2, or scramble shRNA was added into serum-free fresh culture medium at DIV6, and 48 hr after incubation with virus, the culture medium collected before infection was replaced. For *in vivo* experiments, shRNA#1 or shRNA#2 was delivered through bilateral intraventricular injection to 3-week-old mice and the behavioral tasks were performed 2 weeks later.

### 2.4. Cell Staining

For the neuron staining, we followed the methods of Zhang et al. [18]. For colocalization, cortical neurons on coverslips were fixed with 4% paraformaldehyde for 10 min after a brief rinse in prewarmed ECS at room temperature, then permeabilized, and blocked through incubating in PBS containing 0.5% Triton X-100 and 5% BSA for 0.5 hr. Then, the neurons were incubated with primary antibodies to SIL1 (rabbit anti-SIL1, Abcam) and synaptophysin (mouse anti-synaptophysin, Abcam) or SD95 (mouse anti-PSD95, Abcam) in PBS containing 0.5% Triton X-100 overnight at 4°C. After rinsing in PBS containing 0.5% Triton X-100 3 times, neurons were incubated with both Alexa 488-conjugated secondary antibody and Alexa 546-conjugated secondary antibody (donkey anti-rabbit or mouse secondary antibody, Abcam) for 1 h at room temperature. After rinsing with PBS three times, neurons were examined under a 60x, 1.4 numerical aperture oil-immersion objective on an Olympus confocal microscope.

### 2.5. PSD Fraction Separation

For the Western blot analysis of the PSD fraction, we followed the methods of Zhang et al. [18]. After rinsing twice with prewarmed ECS, the DIV14 neurons were homogenized in buffer A (320 mM sucrose, 10 mM HEPES, pH 7.4) through ultrasonication and centrifuged at 12 000×g for 20 min and the pellet was collected as a crude membrane fraction. The pellet was then dissolved in buffer B (4 mM HEPES, 1 mM EDTA, pH 7.4) and centrifuged at 12 000×g for another 20 min. After centrifugation, the pellet was further dissolved in buffer C (20 mM HEPES, 100 mM NaCl, 0.5% Triton X-100, pH 7.2) and incubated for 15 min at 4°C. The homogenate was then centrifuged at 12 000×g for 20 min, and the pellet was the Triton X-100-insoluble PSD fraction. This pellet was dissolved in buffer D (20 mM HEPES, 0.15 mM NaCl, 1% Triton X-100, 1% deoxycholic acid, 1% SDS, 1 mM DTT, pH 7.5) and incubated at 4°C for 1 hr. After centrifugation at 10 000×g for 15 min, a 4x sample buffer was added to the supernatant and the sample was boiled at 100°C for 10 min for Western blot.

### 2.6. Protein Extraction

For the protein extraction, we followed the methods of Zhang et al. [18]. DIV14 cortical cultures were rinsed three times in ECS, and then 500 *μ*l RIPA buffer was added into each 60 mm dish, and the neurons were then collected and ultrasonicated twice for 8 sec each followed by incubation at 4°C for 2 hr. Homogenized neurons were then centrifuged at 12 000×g for 10 min at 4°C, and a 4x sample buffer was added to the supernatant and boiled at 100°C for 10 min, then used for SDS-PAGE. Adult mouse brain tissue was homogenized in RIPA buffer and then centrifuged at 700×g for 10 min at 4°C; the supernatant was incubated at 4°C for 2 hr and then centrifuged at 12 000×g for 10 min at 4°C. The supernatant was then mixed with a 4x sample buffer and boiled at 100°C for 10 min for Western blot.

### 2.7. Coimmunoprecipitation

For the coimmunoprecipitation, we followed the methods of Zhang et al. [18]. Briefly, after protein extraction from mouse cortical tissue, the protein concentration of the supernatant was adjusted to 1 *μ*g/*μ*l. 10 *μ*g of the corresponding antibody was added to 200 *μ*l of the sample and incubated overnight at 4°C. 45 *μ*l of the sample was collected as total protein (input). Then, 30 *μ*l of solubilized protein A-sepharose beads was added and incubated with the sample for another 2 hr at 4°C. Then, the mixture was rinsed three times with binding buffer and once with binding buffer containing 500 mM NaCl to remove nonspecific proteins. After final centrifugation, the supernatant was collected and the pellet was incubated with a 2x sample buffer and incubated at 90°C for 10 min. The input, supernatant, and pellet were then used for SDS-PAGE. For the input and supernatant, 15 *μ*l of 4x sample buffer was added to 45 *μ*l of the sample and then incubated at 90°C for 10 min before being used for Western blot.

### 2.8. cLTP Stimulation

For the cLTP stimulation, we followed the methods of Zhang et al. [18]. DIV14 cultured cortical neurons were rinsed twice in prewarmed ECS and then incubated in buffer A (0.5 *μ*M TTX, 1 *μ*M strychnine, 20 *μ*M bicuculline in ECS, pH 7.4) for 5 min and then in buffer B (200 *μ*M glycine in buffer A) for another 5 min. After rinsing in ECS for 5 min once, neurons were incubated in buffer A for another 20 min. For the control treatment, buffer B was replaced by ECS. The protein sample was then prepared as described in Protein Extraction and used for Western blot.

### 2.9. Biotinylation

For the biotinylation experiment, we followed the methods of Zhang et al. [18]. DIV14 cultured cortical neurons in a 60 mm culture dish were rinsed twice using ice-cold PBS with 1 mM MgCl_2_ and 0.5 mM CaCl_2_ and then incubated with PBS with 1 mg/ml sulfo-NHS-LC-biotin (Thermo Fisher Scientific) for 30 min at 4°C. 500 *μ*l PBS with 100 *μ*M glycine was then added to each 60 mm dish to quench the biotin binding for 15 min at 4°C. Neurons were then lysed in ice-cold RIPA buffer (0.1% SDS, 0.5% sodium deoxycholate, and 1% Triton X-100) after rinsing twice with ice-cold PBS and incubated at 4°C for another 30 min. Cells were concentrated at 12 000×g for 10 min, and the supernatant was incubated with NeutrAvidin beads (Thermo Fisher Scientific) for 2 hr at 4°C. After incubation, the beads were then washed three times with RIPA buffer and proteins were mixed with a 2x sample buffer and boiled at 100°C for 10 min before Western blot.

### 2.10. Barnes Maze Task

For the Barnes maze task, the mice have one habituation trial where it is guided to the shelter manually by the experimenter immediately after being positioned on the platform. This is followed by a 4-day acquisition period, where each day, the mice were allowed to navigate freely in the maze for three minutes, after which it is again manually guided to the shelter if it does not reach it during the exploration period. The last stage of the assay is one probe trial on the 5th day where the animal explores the maze with all holes closed. Learning and long-term memory are quantified by the time taken to find the shelter during the acquisition period and by the time spent near the closed shelter hole in the probe trial. Direct intraventricular injection of lentivirus of shRNA#1, shRNA#2, or scramble shRNA was performed when mice were 3 weeks old, and the Barnes maze task was performed 4 weeks later.

### 2.11. Statistical Analysis

All data were analyzed using GraphPad prism 5 software and are represented as mean ± SEM. Data from multiple groups were quantified using one-way ANOVA, and comparisons of two groups were quantified by unpaired *t*-test.

## 3. Results

### 3.1. Result 1: The Expression Profile of SIL1 *In Vivo* and *In Vitro*

**Figure 1:**
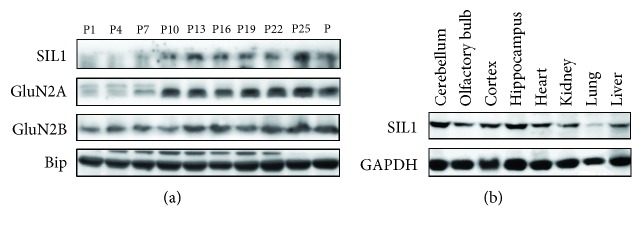
Expression of SIL1 in tissues. (a) Whole lysate of the cerebral cortex at various developmental stages (every 4 days since postnatal day 1 and the last sample was the maternal mice) was subjected to Western blot, and the expression of SIL1, GluN2A, GluN2B, and GAPDH was examined. (b) Whole lysate of adult mouse tissues was examined for the SIL1 expression, including the cerebellum, olfactory bulb, cortex, hippocampus, heart, kidney, lung, and liver.

In our previous study, we found that Bip is required for the dynamic synaptic insertion of the GluN2A-containing NMDA receptor which is crucial for fear conditioning [18]. SIL1 is a cofactor of Bip and has been found to play important roles in the central nervous system [9, 19, 20]. Therefore, in this study, we wanted to investigate the potential role of SIL1 in regulating GluN2A trafficking. We first examined the developmental expression pattern of SIL1 in the cortex during development and its expression in adult mouse tissues. In consistency with former studies [19], we found that the level of SIL1 was very low during early development in mouse cortical tissues (Figure 1(a)) and this is different from Bip which is highly expressed throughout development [21]. Furthermore, we found that the expression pattern of SIL1 was very similar with GluN2A that both proteins underwent a sudden increase between postnatal 7 and 10 days (Figure 1(a)). Thereafter, SIL1 maintained a relatively high level in the brain especially in the hippocampus and cerebellum compared to other tissues (Figure 1(b)). It is well acknowledged that the NMDA receptor has a subtype switch from GluN2B dominant to GluN2A dominant in early development during which the level of GluN2B maintains constant while GluN2A increases rapidly, and it is necessary for the proper function of the central nervous system. Therefore, this result suggested that SIL1 may participate in the developmental subtype switch of the NMDA receptor.

**Figure 2:**
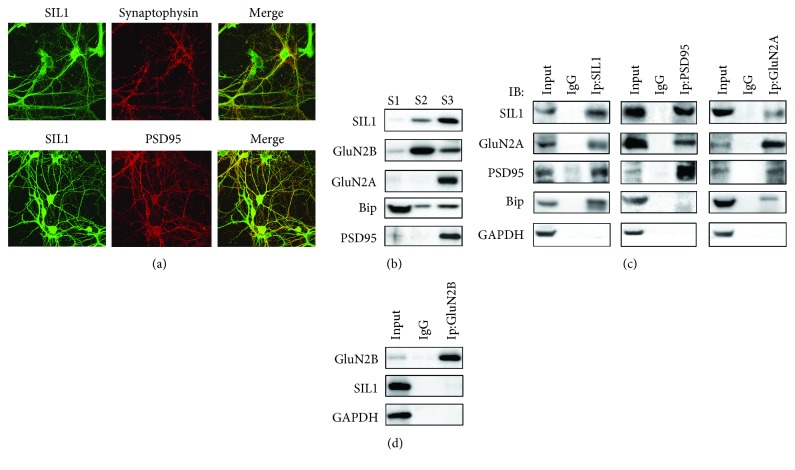
Expression of SIL1 in cultured cortical neurons. (a) Cellular localization of SIL1 was examined in cultured cortical neurons at DIV14. SIL1 was immunostained with anti-SIL1 antibody (green), and synaptophysin or PSD95 was immunostained with anti-synaptophysin or anti-PSD95 antibody (red). Scale bar equals to 10 *μ*m. (b) Expression of SIL1 in different subneuronal compartments of the adult mouse cortex tissue was investigated. S1 indicated extrasynaptic membrane fraction, S2 indicated Triton X-100-soluble synaptic faction, and S3 indicated Triton X-100-insoluble PSD fraction. (c, d) Interactions between SIL1 and GluN2A, PSD95, Bip, and GluN2B were examined by coimmunoprecipitation. Anti-SIL1, anti-PSD95, anti-GluN2A, and anti-GluN2B antibodies were used to precipitate the other proteins. GAPDH was used as a negative control.

Next, we examined the distribution of SIL1 in DIV14 cultured cortical neurons. The rabbit anti-SIL1 primary antibody and Alexa 488 conjunct anti-rabbit secondary antibody were used to show the distribution of SIL1, and mouse anti-PSD95 or synaptophysin and Alexa 594 conjunct anti-mouse secondary antibody were used to show PSD95 or synaptophysin. We found SIL1 to be extensively expressed in neurons; strong fluorescence could be detected both in soma and in extrusions. Besides, the partial colocalization of SIL1 with PSD95 suggested a localization of SIL1 in the distal dendrite (Figure 2(a)), and therefore, we further evaluated whether SIL1 is expressed within the synapse. We separated the extrasynaptic membrane fraction (S1), Triton X-100-soluble synaptic faction (S2), and Triton X-100-insoluble PSD fraction (S3) from the adult mouse cortex. In consistency with our former study [18], we found Bip to be mainly accumulated at the extrasynaptic membrane fraction and there was also a moderate portion of Bip in the PSD fraction (Figure 2(b)). However, the distribution of SIL1 was different from Bip but similar with GluN2A and GluN2B in which SIL1 mainly accumulated in the PSD fraction while a small amount could also be seen in the Triton X-100-soluble synaptic fraction (Figure 2(b)). Because SIL1 is an ER-resident protein, we assume that its attaching to the postsynaptic membrane depends on its binding with other synaptic proteins; thus, we evaluated the interaction of SIL1 with PSD95 and GluN2A through coimmunoprecipitation (CO-IP). We found mutual interaction between SIL1 and PSD95, as well as GluN2A (Figure 2(c)). Moreover, SIL1 had a stronger interaction with PSD95 compared to GluN2A (Figure 2(c)) while it had no interaction with GluN2B (Figure 2(d)). These results showed that SIL1 is distributed broadly in cortical neurons, and similar with GluN2A, a large portion of SIL1 is located in the postsynaptic compartment. Altogether, these data suggested a relationship between SIL1 and GluN2A, and a possible role of SIL1 is in the trafficking of GluN2A.

### 3.2. Result 2: Silencing of SIL1 Inhibited the Developmental Expression of GluN2A but Not Plasticity-Induced Transient Trafficking of GluN2A

**Figure 3:**
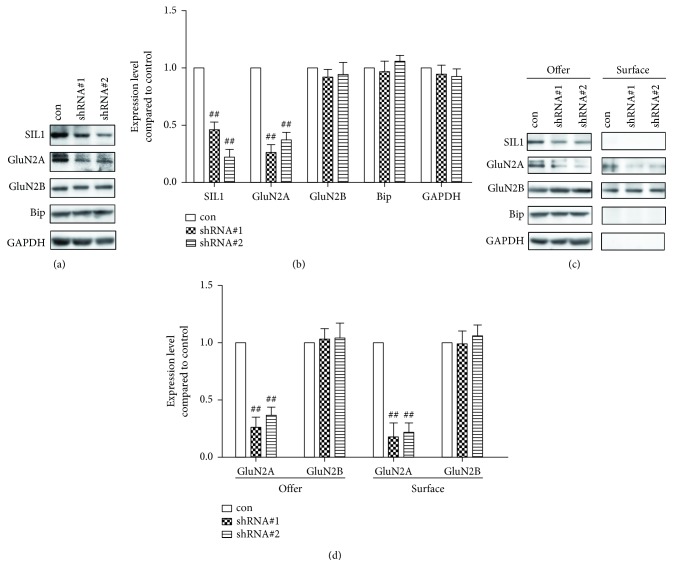
Expression of GluN2A was diminished after SIL1 silencing. (a) Two silencing vectors (shRNA#1 and shRNA#2) targeting SIL1 were designed and transfected into cultured cortical neurons at DIV6 through lentivirus transfection for two-day incubation, and whole cell lysates were harvested at DIV14. The scramble shRNA was used as the control. The expression of SIL1, GluN2A, GluN2B, and Bip was examined by Western blot. (b) Statistical analysis of (a): the expression of each protein was first normalized to GAPDH and then compared with the control. The levels of SIL1 and GluN2A were significantly reduced after shRNA#1 and shRNA#2 were reduced compared to the scramble shRNA-treated control group (SIL1: 46.3% ± 7.2% after shRNA#1 treatment, 26.5% ± 8.5% after shRNA#2 treatment; GluN2A: 26.7% ± 9.1% after shRNA#1 treatment, 37.3% ± 7.2% after shRNA#2 treatment). The levels of GluN2B and Bip were not altered (GluN2B: 92.3% ± 6.5% after shRNA#1 treatment, 94.1% ± 11.6% after shRNA#2 treatment; Bip: 97.4% ± 9.3% after shRNA#1 treatment, 106.2% ± 8.4% after shRNA#2 treatment). (c) The membrane expression of SIL1, GluN2A, GluN2B, and Bip was measured by biotinylation followed by Western blot (surface: membrane fraction; offer: total protein). (d) Statistical analysis of (c): the expression of each protein was first normalized to GAPDH and then compared with the control (GluN2A: 18.6% ± 12.3% after shRNA#1 treatment, 22.5% ± 8.2% after shRNA#2 treatment; GluN2B: 99.3% ± 11.6% after shRNA#1 treatment, 106.4% ± 9.8% after shRNA#2 treatment). All data were presented as mean ± SEM. ^##^*P* < 0.01. *n* = 6 for all treatments.

Since we found that the developmental expression of SIL1 was very similar to that of GluN2A, and SIL1 interacted with GluN2A in cultured cortical neurons, we next investigated whether SIL1 was required for the normal expression of GluN2A. Therefore, we used shRNA to suppress the expression of SIL1. We designed two lentivirus-encoding shRNA targeting two distinct sequences of mouse SIL1 (shRNA#1 and shRNA#2). The cultured cortical neurons were incubated with either shRNA#1 or shRNA#2 for 48 hr from DIV6 and further cultured until DIV14, and then the protein level was evaluated through Western blot. Meanwhile, in the control group, the neurons were incubated with scramble shRNA. We found that both shRNA#1 and shRNA#2 effectively inhibited the expression of SIL1 (Figures 3(a) and 3(b)), as well as GluN2A, while GluN2B was not affected (Figures 3(a) and 3(b)). Next, we measured the membrane expression of GluN2A and GluN2B through surface biotinylation, and consistent with a former result, membrane expression of GluN2A, but not GluN2B, was reduced significantly after SIL1 silencing (Figures 3(c) and 3(d)). These results suggested that SIL1 is required for the normal developmental expression of GluN2A.

**Figure 4:**
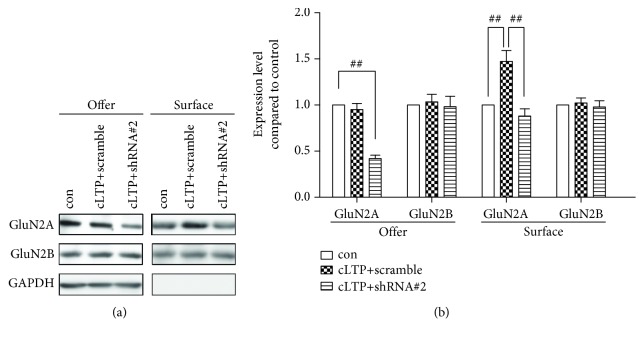
Dynamic trafficking of GluN2A was not affected after SIL1 silencing. (a) Neurons transfected with silencing shRNA or scramble shRNA at DIV6 were subjected to chemical LTP (cLTP) at DIV14 (cLTP+shRNA#2 and cLTP+scramble). Immediately after cLTP treatment, the membrane expression (surface) and total protein level (offer) were assessed by surface biotinylation. Neurons transfected with scramble shRNA without cLTP stimulation were used as the control (con). (b) Statistical analysis of (a): the expression of each protein was first normalized to GAPDH and then compared with the control. The expression of GluN2A reduced after SIL1 silencing (42.7% ± 6.2%, *n* = 6) while the expression of GluN2B was not altered (104.5% ± 7.1%, *n* = 6). In the scramble shRNA-incubated neuron, GluN2A increased after cLTP stimulation (147.2% ± 12.8%, *n* = 6), while in shRNA#2 neurons, GluN2A was also increased after cLTP (88.3% ± 8.9% compared to 42.7% ± 4.2%, *P* < 0.01, *n* = 6) which indicated that the dynamic membrane insertion of GluN2A was not impaired after SIL1 silencing. All data were shown as mean ± SEM. ^##^*P* < 0.01. *n* = 6 for all treatments.

In our former study, we found that Bip regulates the dynamic synaptic insertion of the GluN2A-containing NMDA receptor after receiving plastic stimulation. Since SIL1 is a cofactor of Bip, we further investigated whether SIL1 also regulates the transient trafficking of GluN2A. Thus, we applied chemical long-term potentiation (cLTP) stimuli to the cortical neurons pretreated with silencing shRNA or scramble shRNA and then measured the dynamic membrane insertion of the GluN2A-containing NMDA receptor. In scramble shRNA treatment neurons, the membrane GluN2A was quickly elevated after cLTP as previously reported, while the membrane expression of GluN2A in shRNA#2-treated neurons was significantly lower than scramble shRNA-treated neurons (Figures 4(a) and 4(b)) but was comparable to the neurons without cLTP stimulation (Figures 4(a) and 4(b)). Although we could not directly compare the total and surface expression of GluN2A, it is obvious that the total level of GluN2A in the shRNA#2-treated group is lower than that in the shRNA#2-untreated neurons but the surface fraction of GluN2A in the shRNA#2-treated group is comparable to that in shRNA#2-untreated neurons (Figures 4(a) and 4(b)). Therefore, from this result, we can infer that the dynamic trafficking of GluN2A induced by cLTP stimulation was not fully abolished by SIL1 silencing and the seemingly reduced surface expression of GluN2A was caused by the decrease of overall expression of the protein. Altogether, these results suggested that SIL1 is required for the basic developmental expression of GluN2A but is not necessary for the transient dynamic trafficking of GluN2A in mature neurons.

### 3.3. Result 3: Silencing of SIL1 Impaired the Signaling of the Reelin Signaling Pathway

**Figure 5:**
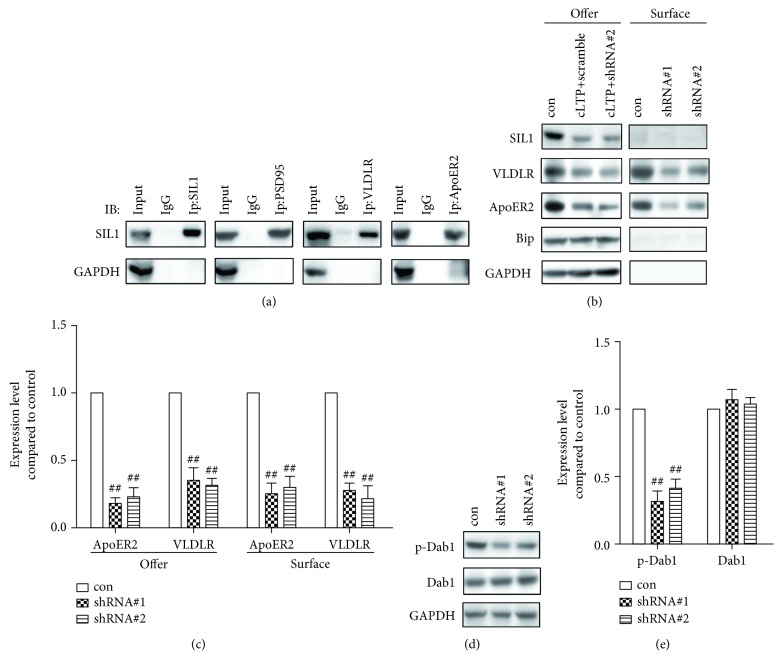
Reelin signaling was impaired after SIL1 silencing. (a) Interactions between SIL1 and PSD95, VLDLR, and ApoER2 were examined by coimmunoprecipitation. Anti-SIL1, anti-PSD95, anti-VLDLR, and anti-ApoER2 antibodies were used to coprecipitate SIL1. GAPDH was used as a negative control. (b) The membrane fraction and total expression of VLDLR and ApoER2 were examined after incubation of neurons with silencing shRNA (shRNA#1 and shRNA#2) or scramble shRNA (con). (c) Statistical analysis of (b): the expression of each protein was first normalized to GAPDH and then compared with the control; both total expression and surface fraction of ApoER2 and VLDLR were significantly reduced (ApoER2 total: 18.2% ± 4.4% after shRNA#1 treatment, 23.6% ± 7.6% after shRNA#2 treatment; ApoER2 surface: 25.8% ± 8.7% after shRNA#1 treatment, 30.5% ± 8.8% after shRNA#2 treatment; VLDLR total: 35.5% ± 9.6% after shRNA#1 treatment, 30.2% ± 8.1% after shRNA#2 treatment; and VLDLR surface: 27.1% ± 6.59% after shRNA#1 treatment, 22.2% ± 9.8% after shRNA#2 treatment). All data were shown as mean ± SEM. ^##^*P* < 0.01. *n* = 6. (d) The phosphorylation of Dab1 after SIL1 silencing (shRNA#1 and shRNA#2) was assessed by antibody targeting phosphorylated Dab1. Neurons treated with scramble shRNA were used as the control (con). (e) Statistical analysis of (d): the phosphorylated Dab1 was first normalized to GAPDH and then to Dab1 and compared with the control (p-Dab1: 36.1% ± 4.8% after shRNA#1 treatment, 44.6% ± 5.2% after shRNA#2 treatment). All data were shown as mean ± SEM. The phosphorylation of Dab1 was significantly inhibited by SIL1 silencing. ^##^*P* < 0.01. *n* = 6 for all treatments.

The above data suggested a novel role of SIL1 in regulating the developmental expression of GluN2A. But it seems that SIL1 was not directly involved in GluN2A maturation and assembly, because if this is the case, then the cLTP-induced GluN2A membrane insertion should also be inhibited after SIL1 silencing. Therefore, we assessed an alternative mechanism that may explain this phenomenon. It has been proved that the Reelin signaling pathway is important for the NMDA receptor composition change during neuron maturation [22, 23, 40]. And also, it was found that the deficiency of cortical neuron migration caused by SIL1 mutation is very similar with that of Reelin knockout mice [19, 24]. Thus, we speculate that silencing of SIL1 might have impaired the Reelin signaling pathway and in turn affected the expression of GluN2A. Reelin is an extracellular matrix protein and functions on neurons through both its receptors, VLDLR and ApoER2 [25]. Therefore, we first examined whether SIL1 was involved in the expression of VLDLR and ApoER2. Through coimmunoprecipitation, we identified a strong interaction between SIL1 and VLDLR as well as ApoER2 (Figure 5(a)). Furthermore, after SIL1 silencing, both the total amount and surface expression of VLDLR and ApoER2 were strongly reduced (Figures 5(b) and 5(c)). These results suggested that the expression of the two main receptors of Reelin signaling depended on SIL1. Because previous studies have shown that the activation of the Reelin signaling pathway is required for the developmental expression of GluN2A [23, 40], we further measured the phosphorylation of Dab1, which is a prominent hub molecule of Reelin signaling and has been known to regulate synaptic maturation [40]. As a result, the phosphorylation level but not the total amount of Dab1 significantly decreased after silencing of SIL1 (Figures 5(d) and 5(e)). The above data showed that SIL1 was required for the expression of both Reelin receptors and therefore played vital roles in the activation of the Reelin signaling pathway.

### 3.4. Result 4: Silencing of SIL1 Impaired the Spatial Memory Which Was Rescued by Mimic Phosphorylation Dab1 Peptide

**Figure 6:**
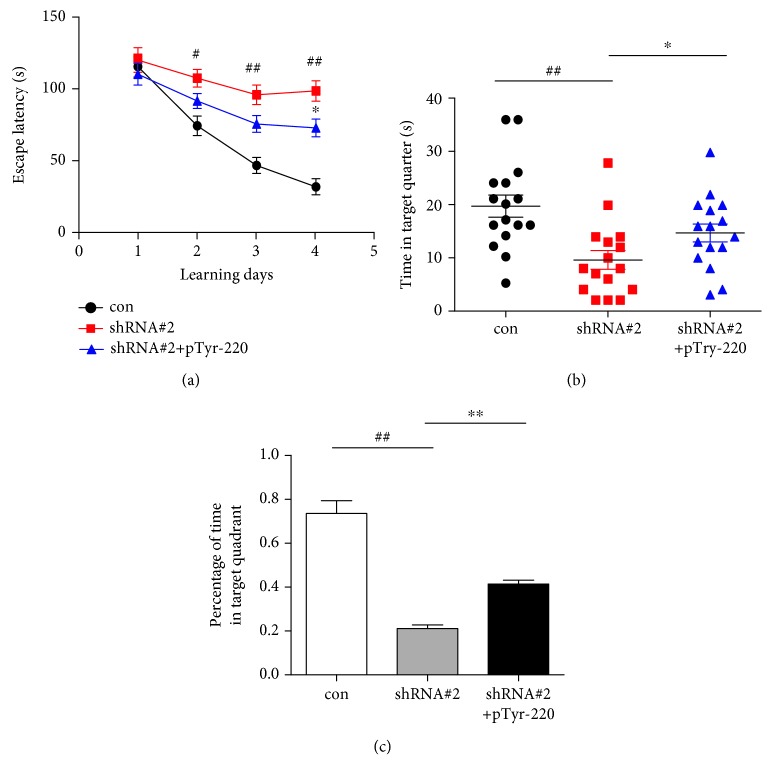
Spatial memory formation of young mice was impaired after SIL1 silencing. (a) Mice were subjected to bilateral intraventrical injection of lentivirus-encoding shRNA#2 at 3 weeks old followed or not followed by daily intraperitoneal injection of mimic phosphorylation peptide of Dab1 (pTyr-220). Barnes maze task was conducted 2 weeks later to assess the spatial learning of mice. The escape latency was measured from day 1 to day 4 of the acquisition period (con: day 1: 115.5 ± 7.3 s, day 2: 73.8 ± 6.9 s, day 3: 45.9 ± 5.6 s, and day 4: 30.7 ± 5.7 s, *n* = 16; shRNA#2: day 1: 120.2 ± 8.7 s, day 2: 107.4 ± 6.2 s, day 3: 95.7 ± 6.9 s, and day 4: 98.4 ± 7.2 s, *n* = 16; and shRNA#2+pTyr-220: day 1: 110.2 ± 7.7 s, day 2: 91.3 ± 5.2 s, day 3: 75.1 ± 5.9 s, and day 4: 72.3 ± 6.2 s, *n* = 16). All data were shown as mean ± SEM. ^#^*P* < 0.05 and ^##^*P* < 0.01 for the shRNA#2 treatment compared to the control; ^∗^*P* < 0.05 for shRNA#2 compared to shRNA#2+pTyr-220. (b) The time that mice spent in the target quarter was measured in the probe trial (con: 19.6 ± 2.1 s, shRNA#2: 9.6 ± 1.8 s, and shRNA#2+pTyr-220: 14.75 ± 1.7 s, *n* = 6). ^##^*P* < 0.01 for shRNA#2 compared to the control and ^∗∗^*P* < 0.01 for shRNA#2 compared to shRNA#2+pTyr-220. *n* = 16 and *N* = 3. (c) The percentage of time that mice spent in the target quarter was measured in the probe trial (con: 73.7% ± 5.6%, shRNA#2: 20.7% ± 2.0%, and shRNA#2+pTyr-220: 41.2% ± 2.0%). All data were shown as mean ± SEM. ^##^*P* < 0.01 for shRNA#2 compared to the control and ^∗^*P* < 0.05 for shRNA#2 compared to shRNA#2+pTyr-220. *n* = 16 and *N* = 3.

Sil1 is the only gene found to be responsible for the MSS which has a major symptom of mental retardation. Previous studies have shown that SIL1 silencing caused a delayed migration of neurons during neocortex development which is similar to Reelin knockout mice [19]. Our data also showed that SIL1 silencing resulted in an inactivation of Reelin signaling and obstructed synaptic NMDA receptor maturation. Other studies have shown that Reelin could modulate learning and memory by modulating NMDA receptor expression and function [40]. These evidence supported that the mental retardation caused by mutation of Sil1 may associate with malfunction of Reelin signaling and the subsequent abnormal expression of the NMDA receptor. Therefore, we examined the spatial memory formation of young mice after SIL1 silencing. The lentivirus-encoding shRNA#2 or scramble shRNA was administered to 3-week-old mice through bilateral ventricular injection. And the Barnes Maze task was performed 2 weeks later to examine the spatial learning of the mice. And we found that the escape latency of the SIL1 silencing mice was significantly longer than that of the control mice in the acquisition period since day 2 (Figure 6(a)) and the time that mice spent in the target quarter as well as the percentage of time in the target quarter in the probe trial is significantly reduced (Figures 6(b) and 6(c)). Since MSS patients show early onset cataract and muscle atrophy, we evaluated the performance of SIL1-silenced mice in two other behavior tasks, the beam-walking task and the rotarod task. The SIL1-deficient mice showed similar latency time compared to scramble shRNA-treated mice (Supplemental Fig. [Supplementary-material supplementary-material-1] and [Supplementary-material supplementary-material-1]), and this result ruled out the possibility that the impaired eyesight or reduced muscle coordination caused the low performance of mice in the Barnes maze task. In order to further prove the involvement of Reelin signaling in SIL1-mediated spatial learning, we used a mimic phosphorylation peptide of Dab1, pTyr-220, which is able to activate the Reelin signaling pathway [26]. We found that daily intraperitoneal injection of pTyr-220 after SIL1 silencing partly rescued the impaired performance of mice in the Barnes maze task (Figures 6(a) and 6(b)). In addition, the expression of GluN2A could also be rescued by pTyr-220 (Supplemental Fig. [Supplementary-material supplementary-material-1] and [Supplementary-material supplementary-material-1]). Altogether, these data provided direct evidence of SIL1 functioning in spatial learning which depended on Reelin signaling and was associated with its role in regulating the developmental maturation of the NMDA receptor.

## 4. Discussion

Sil1 was identified as the causal gene of Marinesco-Sjӧgren Syndrome by two independent research groups in 2005 [8, 9]. Mental retardation is one of the major symptoms of MSS which indicated that SIL1 might play important roles in neurons. In this study, we intended to explore the molecular mechanism of SIL1 in regulating the development of the central nervous system.

We first detected the expression of SIL1 in brain tissues and neurons. We found that SIL1 expression was quite low during the early development which was consistent with other studies [18]. We also found that SIL1 significantly increased its expression among postnatal 7-10 days which was highly similar with the developmental expression pattern of GluN2A. In our previous study, we found that Bip mediated the subunit-selective increase of the GluN2A-containing NMDA receptor which made it a candidate for regulating the maturational change of the NMDA receptor subtype. However, Bip maintains constantly high expression throughout development which seems contradictory to a role in development and also suggested that other related proteins may be involved. Comparing to Bip, SIL1 possesses more characters that fit this function. Firstly, like GluN2A, SIL1 mainly accumulated in the PSD fraction of neurons and had direct interaction with PSD95. Secondly, SIL1 showed similar developmental expression with GluN2A. As a tetramer, each NMDA receptor is composed of two structural subunits of GluN1 and two functional subunits of GluN2 or GluN3. The subunit composition determines the function of different NMDA receptor subtypes; among them, GluN2A and GluN2B are two major types expressed in the neocortex and hippocampus [27–29]. A variety of studies have proved that the GluN2A-containing NMDA receptor is gradually becoming the dominant subtype during development which is mainly caused by the increase of GluN2A [30–32]. And as a result, the threshold for LTP is raised and this is the prerequisite for synaptic pruning and fine tuning of the neuronal circuit [33]. This shift of subtype dominancy is regarded as a symbol of synaptic maturation and is believed to be regulated by sensory stimulus [34, 35], but its molecular mechanism is not fully uncovered yet. Currently, it is known that the phosphorylation of the GluN2B PDZ domain by CK2 may participate in the synaptic clearance of GluN2B [36] and the repressor element 1-silencing transcription factor (REST) could also decrease the GluN2B expression though epigenetic modification of the gene of GluN2B [37]. And for GluN2A, it is believed that the extracellular matrix is very important for its developmental expression, especially the Reelin signaling pathway. Inhibition of the Reelin signaling pathway tempers the GluN2A/GluN2B switch [38, 39] and also impairs the learning and memory [40]. Consistently, we found in our study that SIL1 was not directly involved in the transient dynamic trafficking of GluN2A in mature neurons but it could regulate the developmental increase of GluN2A through the Reelin signaling pathway. After SIL1 silencing, the expression of both Reelin receptors decreased and the phosphorylation of their downstream hub molecule Dab1 was inhibited. According to the previous studies, this will in turn obstruct the expression of GluN2A and affect the learning ability of the mice [23, 38, 40, 41] as we have found in our current study. More importantly, we found that the mimic phosphorylation peptide of Dab1 was able to partially rescue the expression of GluN2A and the spatial learning ability of the mice. Therefore, our data suggested that the functional deficiency of SIL1 could impair spatial learning of mice because of the abnormal expression of GluN2A induced by malfunction of Reelin signaling.

Finally, our data combined with other studies indicated that compared to Bip, SIL1 potentially has more particular functions. SIL1 showed cell-type selectivity in the nervous system. The Amyotrophic lateral sclerosis (ALS)-vulnerable FF motor neurons have much less SIL1 compared to the disease-resistant S motor neurons, and this difference is the main reason for the different resistance of the two types of neurons against ALS [20]. And SIL1 also showed substrate selectivity. For example, the normal secretion of antibody in SIL1 knockout mice indicated that SIL1 is not involved in or it is not essential for the antibody assembly in B cells [42] which is a major function of Bip [43, 44]. But SIL1 is required for the insulin secretion from mouse pancreatic beta cells [45]. Actually, there is another nucleotide exchange factor of Bip which is GRP170 [46]. It has been proved that GRP170 interacts with unassembled antibody [47, 48]. Therefore, the two nucleotide exchange factors SIL1 and GRP170 may function separately. In our study, we discovered a novel substrate selection that SIL1 regulated the expression of GluN2A but not its homolog GluN2B. This functional selectivity is consistent with the finding that unlike Bip, the SIL1-mutated mice are viable [17]. It will be interesting to investigate the mechanism of the SIL1 selectivity and its physiological significance in the development of the central nervous system.

## Data Availability

The data used to support the findings of this study are included within the article.

## Supplementary Materials

Supplementary Materials Mice were subjected to bilateral intraventricular injection of lentivirus-encoding shRNA#2 at 3 weeks old followed or not followed by daily intraperitoneal injection of pTyr-220. Mice that received scramble shRNA injection were used as the control (con). Then, the whole cortex lysate was obtained at 5 weeks and GluN2A was measured by Western blot. (B) Statistical analysis of (A). The expression of GluN2A was normalized to GAPDH first and then compared with the control (con) in which group neurons were transfected with scramble shRNA. After SIL1 silencing, GluN2A expression was reduced significantly (16.0% ± 4.0%, *P* < 0.01, and *n* = 6) but rescued after applying pTyr-220 (87.5% ± 6.2%, *P* < 0.01 compared to shRNA#2 treatment neurons and *P* = 0.07 compared to the control). All data were shown as mean ± SEM. ^##^*P* < 0.01. *n* = 6 and *N* = 3 for all treatments.
