# Supplementary material for: Functional Role of SIL1 in Neurodevelopment and Learning
Source: Neural Plast. 2019 Aug 18;2019:9653024. doi: 10.1155/2019/9653024 (PMC6720716; doi:10.1155/2019/9653024)
Supplement: Supplementary Materials — Mice were subjected to bilateral intraventricular injection of lentivirus-encoding shRNA#2 at 3 weeks old followed or not followed by daily intraperitoneal injection of pTyr-220. Mice that received scramble shRNA injection were used as the control (con). Then, the whole cortex lysate was obtained at 5 weeks and GluN2A was measured by Western blot. (B) Statistical analysis of (A). The expression of GluN2A was normalized to GAPDH first and then compared with the control (con) in which group neurons were transfected with scramble shRNA. After SIL1 silencing, GluN2A expression was reduced significantly (16.0% ± 4.0%, P < 0.01, and n = 6) but rescued after applying pTyr-220 (87.5% ± 6.2%, P < 0.01 compared to shRNA#2 treatment neurons and P = 0.07 compared to the control). All data were shown as mean ± SEM. ##P < 0.01. n = 6 and N = 3 for all treatments. [file 9653024.f1.pdf]

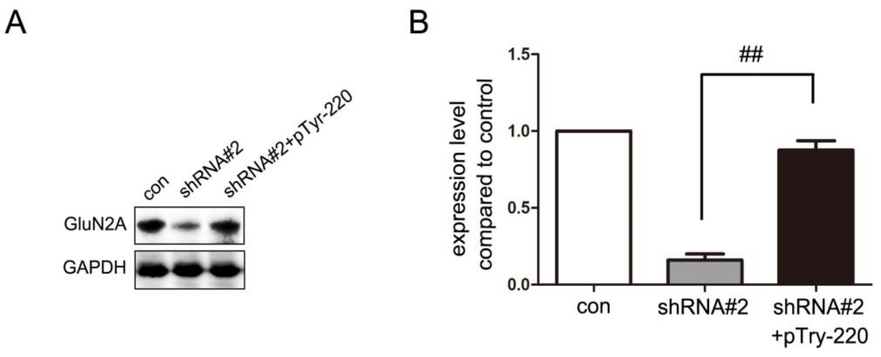

Supplemental Fig. 1. The expression of GluN2A was rescued by pTry-220.

(A). Mice were subjected to bilateral intraventrical injection of lentivirus encoding shRNA#2 at 3-week old followed or not followed by daily intraperitoneal injection of pTyr-220. Mice received scramble shRNA injection were used as control (con). Then the whole cortex lysate was obtain at 5-week and GluN2A was measured by western blot. (B). Statistical analysis of (A). The expression of GluN2A was normalized to GAPDH first and then compared with control (con) in which group neurons were transfected with scramble shRNA. After SIL1 silencing, GluN2A expression was reduced significantly (16.0%±4.0%, $P<0.01$, $n=6$) but rescued after applying pTyr-220 (87.5%±6.2%, $P<0.01$ compared to shRNA#2 treatment neurons, $p=0.07$ compared to control). All data were shown as mean±SEM. ## $P<0.01$. $n=6$, $N=3$ for all treatments.

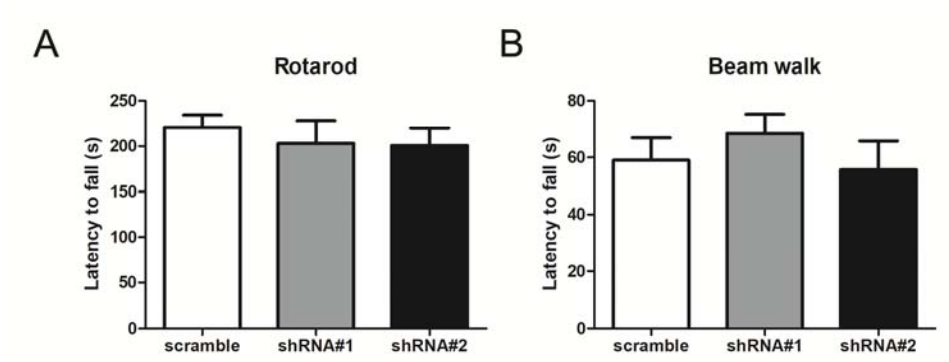

Supplemental Fig. 2. The performance of SIL1 silencing mice in rotarod and beam walk tasks was not impaired.

(A,B). Mice were subjected to bilateral intraventrical injection of lentivirus encoding shRNA#1 or shRNA#2 at 3-week old, and the behavior tasks were conducted at 5-week old. Mice received scramble shRNA injection was used as control. The SIL1 silencing mice showed similar latency to fall with control mice in Rotarod task (scramble: 220.7±13.6s; shRNA#1: 203.0±25.12s; shRNA#2: 200.8±19.1s) and in Beam Walk task (scramble: 59±8.0s; shRNA#1: 68.5±6.7s;

shRNA#2: 55.7±10.1s) . All data were shown as mean±SEM. n= 6 to 8, N=3 for all treatments.
